# Machine learning-based prediction of vitamin D deficiency: NHANES 2001-2018

**DOI:** 10.3389/fendo.2024.1327058

**Published:** 2024-02-16

**Authors:** Jiale Guo, Qionghan He, Yehai Li

**Affiliations:** ^1^ Department of Orthopedics, Chaohu Hospital of Anhui Medical University, Hefei, China; ^2^ Department of Infection, Chaohu Hospital of Anhui Medical University, Hefei, China

**Keywords:** machine learning, vitamin D deficiency, clinical decision rules, nutrition surveys, public health

## Abstract

**Background:**

Vitamin D deficiency is strongly associated with the development of several diseases. In the current context of a global pandemic of vitamin D deficiency, it is critical to identify people at high risk of vitamin D deficiency. There are no prediction tools for predicting the risk of vitamin D deficiency in the general community population, and this study aims to use machine learning to predict the risk of vitamin D deficiency using data that can be obtained through simple interviews in the community.

**Methods:**

The National Health and Nutrition Examination Survey 2001-2018 dataset is used for the analysis which is randomly divided into training and validation sets in the ratio of 70:30. GBM, LR, NNet, RF, SVM, XGBoost methods are used to construct the models and their performance is evaluated. The best performed model was interpreted using the SHAP value and further development of the online web calculator.

**Results:**

There were 62,919 participants enrolled in the study, and all participants included in the study were 2 years old and above, of which 20,204 (32.1%) participants had vitamin D deficiency. The models constructed by each method were evaluated using AUC as the primary evaluation statistic and ACC, PPV, NPV, SEN, SPE, F1 score, MCC, Kappa, and Brier score as secondary evaluation statistics. Finally, the XGBoost-based model has the best and near-perfect performance. The summary plot of SHAP values shows that the top three important features for this model are race, age, and BMI. An online web calculator based on this model can easily and quickly predict the risk of vitamin D deficiency.

**Conclusion:**

In this study, the XGBoost-based prediction tool performs flawlessly and is highly accurate in predicting the risk of vitamin D deficiency in community populations.

## 1 Introduction

Vitamin D is a unique fat-soluble vitamin, and as it is produced primarily through exposure of human skin to sunlight, few foods contain natural vitamin D (1). Its main role in humans is to increase the absorption of calcium and phosphate to mineralize the bones (2). In children, vitamin D deficiency leads to growth retardation and rickets (3). In adults, vitamin D deficiency can lead to osteochondrosis and osteoporosis (3). Vitamin D deficiency and its health consequences first gained attention with the industrialization of Northern Europe. As research progressed, vitamin D deficiency was also found to be strongly associated with the development of diabetes (4), sarcopenia (5), psychiatric disorders (6), autoimmune diseases (7), cardiovascular diseases (8), and tumors (9). Because of the role of vitamin D in the antiviral immune response (10, 11), vitamin D-related studies have gained more attention since the COVID-19 pandemic. Vitamin D levels have also been shown to be associated with the prevention and prognosis of COVID-19 (12–14). Vitamin D deficiency has now been defined as a pandemic. As an important part of public health, identifying vitamin D deficiency is vital. However, a single measurement of vitamin D costs £9.86 and between 70.4% and 77.5% of tests are likely to be inappropriate (15). Testing for vitamin D in all populations does not appear to be appropriate. An Endocrine Society Clinical Practice Guideline recommends screening for vitamin D in people at risk for deficiency; they do not recommend screening for vitamin D in people who are not at risk (16). The use of prediction tools to identify patients at high risk of vitamin D deficiency is necessary. As of now, there are no prediction tools for predicting vitamin D risk in the general community population.

Machine learning is one of the fastest growing technology areas today and is widely used to enable evidence-based decision making in industries such as healthcare, manufacturing, and education (17). Machine learning is primarily based on large datasets to develop robust risk models and predict the type of person being studied (18, 19). Prediction tools developed using machine learning can be a good predictor of vitamin D deficiency risk in participants. The purpose of this study was to construct a prediction tool to predict participants’ risk of vitamin D deficiency using a machine learning method based on data that can be easily collected in a general community population.

## 2 Materials and methods

### 2.1 Data sources and study population

Data for this study were obtained from the National Health and Nutritional Examination Surveys (NHANES), a population-based, cross-sectional survey study conducted in two-year cycles since 1999 to assess the health and nutritional status of adults and children in the United States. Serum 25(OH)D as a good biomarker for evaluating vitamin D status was used in this study as a laboratory test to determine vitamin D deficiency (20). The definition of vitamin deficiency used in this study was 25(OH)D < 50 nmol/L as recommended by an Endocrine Society Clinical Practice Guideline (16). Data from NHANES 2001-2018 containing 25(OH)D measurements were included in this study. In particular, serum 25(OH)D data from NHANES 2001-2006 were determined by the radioimmunoassay (RIA) method, which, due to excessive methodological bias and inaccuracy, was switched to liquid chromatography-tandem mass spectrometry (LC-MS/MS), a method that has better specificity and sensitivity, in the follow-up to NHANES 2007-2018 (21). Whereas serum 25(OH)D data from NHANES 2001-2006 have been converted to 25(OH)D measurements from equivalent LC-MS/MS methods by using regression.

For simplicity and ease of use of the model, only information that could be obtained in the community through a simple interview was included as variables for instrument development: gender, age, race, total number of people in the Household (H.Size), household income to poverty income ratio (H.PIR), body mass index (BMI), whether or not someone smokes in the household (H.Smoke), past 30-day milk product consumption (Milk), diabetes. Race is categorized as Mexican American, Non-Hispanic White, Non-Hispanic Black, Other Hispanic, or Other Race. For H.Size over 7 or more defined as 7. For H.PIR more than 5 is defined as 5. For the past 30-day milk product consumption, four frequencies were used to distinguish between never, rarely, sometimes, and often, with never meaning never drinking milk; rarely meaning less than once a week; sometimes meaning once a week or more but less than once a day; and often meaning once a day or more.

The data analyzed in this study were obtained from NHANES and did not require additional ethical review by the investigator’s affiliated institution. NHANES has received approval from the National Center for Health Statistics (NCHS) Research Ethics Review Board.

### 2.2 Statistical analysis

Normally distributed continuous variables are expressed as mean ± standard deviation, non-normally distributed continuous variables as median (interquartile range), and categorical variables as percentages. Continuous variables were analyzed with the Independent Student’s t-test or Mann-Whitney U analysis; categorical variables were analyzed with the chi-square test or Fisher’s test. All statistical analyses were realized based on the “CBCgrps” package in R software.

### 2.3 Model construction, evaluation and validation

Data from the NHANES database for nine cycles from 2001-2018 were included for analysis. The included data were randomly divided into training and validation sets in the ratio of 70:30. We used the extracted variables as machine learning features for analysis. Six machine learning algorithms, Gradient Boosting Machine (GBM), Logistic Regression (LR), Neural Network (NNet), Random Forest (RF), Support Vector Machine (SVM), and eXtreme Gradient Boosting (XGBoost), were used to construct the classification model. Ten 10-fold cross validation resampling was used to ensure stability and reproducibility of model performance. Receiver operating characteristic (ROC) curves were plotted to evaluate the discriminative performance of the model, and the area under the curve (AUC) of the ROC curve was calculated. The AUC value was used as the main statistical indicator to evaluate the predictive performance of the model. To evaluate the predictive performance of the model more comprehensively, this study also reports accuracy (ACC), positive predictive value (PPV), negative predictive value (NPV), sensitivity (SEN), specificity (SPE), F1 score, Matthews correlation coefficient (MCC). The closer these statistics are to 1 the better the predictive performance of the model. Kappa values are used to determine whether the model’s results are consistent with actual results. The Kappa value is between -1 and +1, the closer the Kappa value is to 1, the better the consistency is, and if it is greater than 0.75, the consistency is excellent. The Brier Score combines the differentiation and calibration of the model and is used to evaluate the overall performance of the model, and the closer the Brier Score is to 0, the closer the predicted value is to the actual value (22). Decision curve analysis (DCA) is used to assess the clinical utility of models in decision making (23). The best machine learning predictive model was selected using AUC statistic value as the main statistic combined with various statistical indicators. Shapely Additive exPlanations (SHAP) values were used to interpret the best machine learning models (24). In addition, for the best machine learning models, an online web calculator is further constructed to facilitate the use of the models.

All statistical analyses, model construction and validation in this study were based on R software (version 4.1.3).

## 3 Results

**Figure 1:**
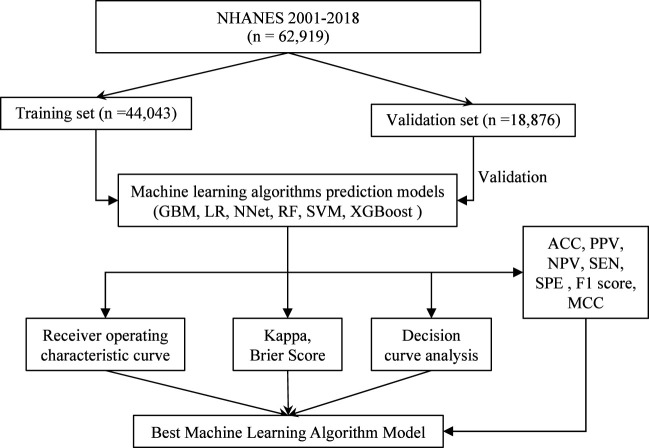
Flowchart of data screening and analysis. NHANES, National Health and Nutritional Examination Surveys; GBM, Gradient Boosting Machine; LR, Logistic Regression; NNet, Neural Network; RF, Random Forest; SVM, Support Vector Machine; XGBoost, eXtreme Gradient Boosting; ACC, accuracy; PPV, positive predictive value; NPV, negative, predictive value; SEN, sensitivity; SPE, specificity; MCC, Matthews correlation coefficient.

**Figure 2:**
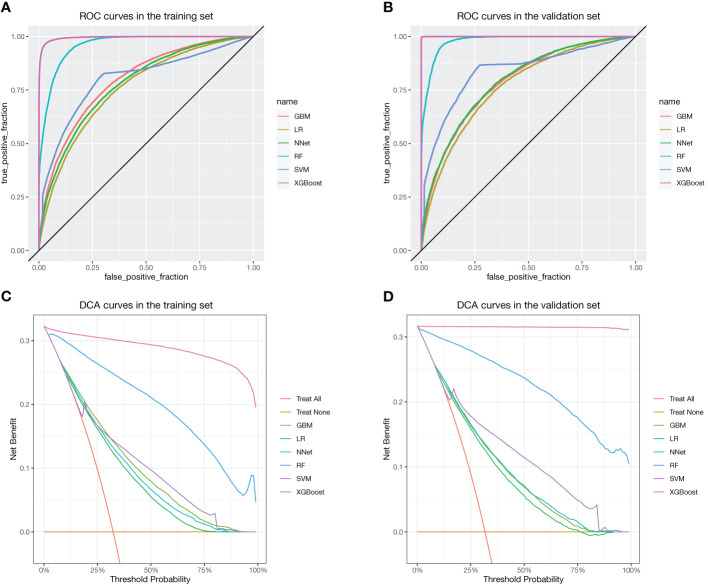
ROC and DCA curves for each method. **(A)** ROC in the training set. **(B)** ROC in the validation set. **(C)** DCA curves in the training set. **(D)** DCA curves in the validation set.

There were 62,919 participants enrolled in the study, all the participants included in the study were 2 years old and above, of which 20,204 (32.1%) participants had vitamin D deficiency. The entire flow of the analysis is shown in the flowchart (**Figure 1**). The included data were randomly divided into training and validation sets in a ratio of 70:30, and the characteristics of the patients in the training set are shown in **Table 1**. The performance of the models constructed by each method was determined by resampling with ten ten-fold cross validation. AUC values were calculated based on the ROC curves (**Figures 2A, B**). The AUC values of GBM, LR, NNet, RF, SVM, and XGBoost in the training set are 0.796, 0.76, 0.778, 0.96, 0.8, and 0.995, respectively; and in the validation set are 0.786, 0.767, 0.79, 0.979, 0.837, and 1, respectively (**Table 2**). The model constructed by the XGBoost method has the best and near-perfect prediction performance in both the training and validation sets. To avoid the bias caused by data imbalance, this study further calculates ACC, PPV, NPV, SEN, SPE, F1 score, and MCC to evaluate the prediction performance of the model more comprehensively, as shown in **Table 2**. XGBoost obtained excellent results on all types of statistical metrics used to evaluate differentiation. The Kappa values of GBM, LR, NNet, RF, SVM, XGBoost in the training set are: 0.407, 0.353, 0.382, 0.745, 0.476, 0.928; and in the validation set are: 0.395, 0.36, 0.38, 0.821, 0.53, 0.997 (**Table 2**). The Brier score values of GBM, LR, NNet, RF, SVM, XGBoost in the training set are: 0.165, 0.178, 0.172, 0.084, 0.166, 0.042 respectively; and in the validation set are: 0.168, 0.175, 0.166, 0.068, 0.154, 0.013 respectively (**Table 2**). The XGBoost method also shows excellent consistency. The DCA curves show that the XGBoost-based model achieves higher net gains than the “all intervention” or “no intervention” strategies over the full range of thresholds, both in the training set (**Figure 2C**) and the validation set (**Figure 2D**). Combined with the various model performance evaluation statistics, the XGBoost-based model has the best and almost perfect performance.

**Table 1:** Characterization of participants in the training set.

Variables	Total (n = 44043)	NVDD (n = 29818)	VDD (n = 14225)	p
Gender, n (%)				< 0.001
Female	22419 (51)	14712 (49)	7707 (54)	
Male	21624 (49)	15106 (51)	6518 (46)	
Age, Median (Q1, Q3)	31 (14, 54)	31 (12, 56)	30 (17, 51)	< 0.001
Race, n (%)				< 0.001
Mexican American	8954 (20)	5636 (19)	3318 (23)	
Non-Hispanic Black	9958 (23)	4191 (14)	5767 (41)	
Non-Hispanic White	17339 (39)	14743 (49)	2596 (18)	
Other Hispanic	3538 (8)	2463 (8)	1075 (8)	
Other Race	4254 (10)	2785 (9)	1469 (10)	
H.Size, n (%)				< 0.001
1	3935 (9)	2635 (9)	1300 (9)	
2	9364 (21)	6673 (22)	2691 (19)	
3	7570 (17)	4964 (17)	2606 (18)	
4	8782 (20)	6030 (20)	2752 (19)	
5	6991 (16)	4750 (16)	2241 (16)	
6	3746 (9)	2462 (8)	1284 (9)	
7	3655 (8)	2304 (8)	1351 (9)	
H.PIR, Median (Q1, Q3)	1.87 (0.99, 3.69)	2.04 (1.05, 3.98)	1.6 (0.88, 3.1)	< 0.001
BMI, Median (Q1, Q3)	25.2 (20.45, 30.2)	24.4 (19.6, 29.2)	26.9 (22.16, 32.49)	< 0.001
H.Smoke, n (%)				< 0.001
No	34722 (79)	23934 (80)	10788 (76)	
Yes	9321 (21)	5884 (20)	3437 (24)	
Milk, n (%)				< 0.001
Never	5329 (12)	3029 (10)	2300 (16)	
Often	22374 (51)	16986 (57)	5388 (38)	
Rarely	5618 (13)	3172 (11)	2446 (17)	
Sometimes	10722 (24)	6631 (22)	4091 (29)	
Diabetes, n (%)				< 0.001
No	40652 (92)	27637 (93)	13015 (91)	
Yes	3391 (8)	2181 (7)	1210 (9)	

NVDD, non-vitamin D deficiency; VDD, vitamin D deficiency; H.Size, total number of people in the Household; H.PIR, household income to poverty income ratio; BMI, body mass index; H.Smoke, whether or not someone smokes in the household; Milk, past 30-day milk product consumption.

**Table 2:** Evaluation metrics of the models constructed by each method.

	Method	AUC	ACC	PPV	NPV	SEN	SPE	F1 score	MCC	KAPPA	Brier score
Tra	GBM	0.796	0.717	0.546	0.849	0.736	0.708	0.627	0.419	0.407	0.165
	LR	0.76	0.685	0.509	0.837	0.728	0.664	0.599	0.368	0.353	0.178
	Nnet	0.778	0.706	0.534	0.836	0.71	0.705	0.61	0.392	0.382	0.172
	RF	0.96	0.882	0.76	0.962	0.928	0.86	0.835	0.754	0.745	0.084
	SVM	0.8	0.747	0.577	0.887	0.808	0.718	0.674	0.494	0.476	0.166
	XGBoost	0.995	0.968	0.937	0.984	0.967	0.969	0.952	0.929	0.928	0.042
Val	GBM	0.786	0.716	0.539	0.842	0.709	0.719	0.612	0.404	0.395	0.168
	LR	0.767	0.689	0.506	0.847	0.74	0.665	0.601	0.378	0.36	0.175
	Nnet	0.79	0.694	0.511	0.865	0.78	0.654	0.618	0.404	0.38	0.166
	RF	0.979	0.919	0.817	0.98	0.96	0.9	0.882	0.828	0.821	0.068
	SVM	0.837	0.772	0.597	0.921	0.866	0.728	0.706	0.554	0.53	0.154
	XGBoost	1	0.999	0.998	0.999	0.998	0.999	0.998	0.997	0.997	0.013

Tra, training set; Val, validation set; AUC, area under the curve; ACC, accuracy; PPV, positive predictive value; NPV, negative predictive value; SEN: sensitivity; SPE: specificity, MCC, Matthews correlation coefficient.

**Figure 3:**
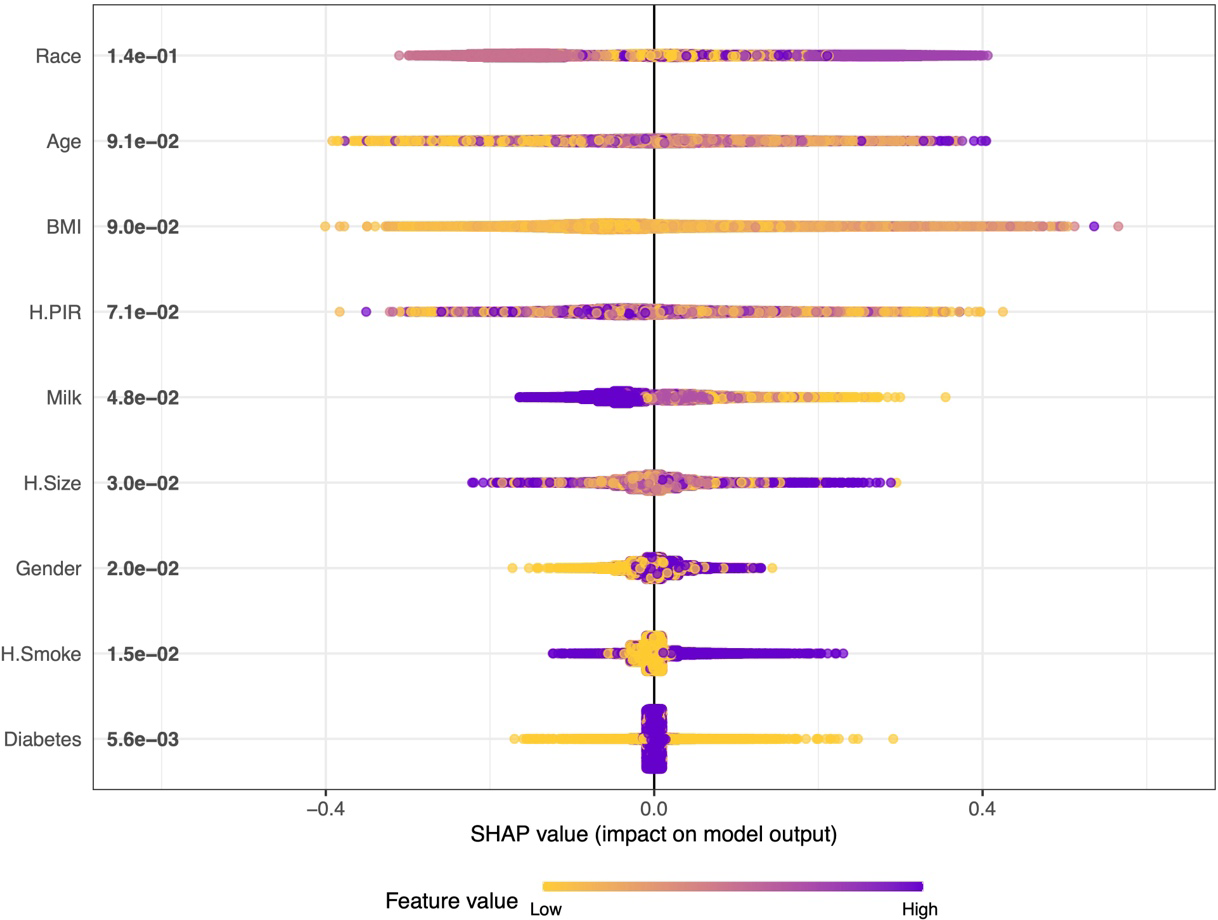
Summary plot of SHAP values for the model constructed by XGBoost algorithm. The horizontal position “SHAP value” indicates whether the impact of the value is associated with a higher or lower prediction, and the color of each SHAP value point indicates whether the observed value is higher (purple) or lower (yellow). The vertical coordinates show the importance of the features, sorted by the importance of the variables in descending order, with the upper variables being more important to the model.

**Figure 4:**
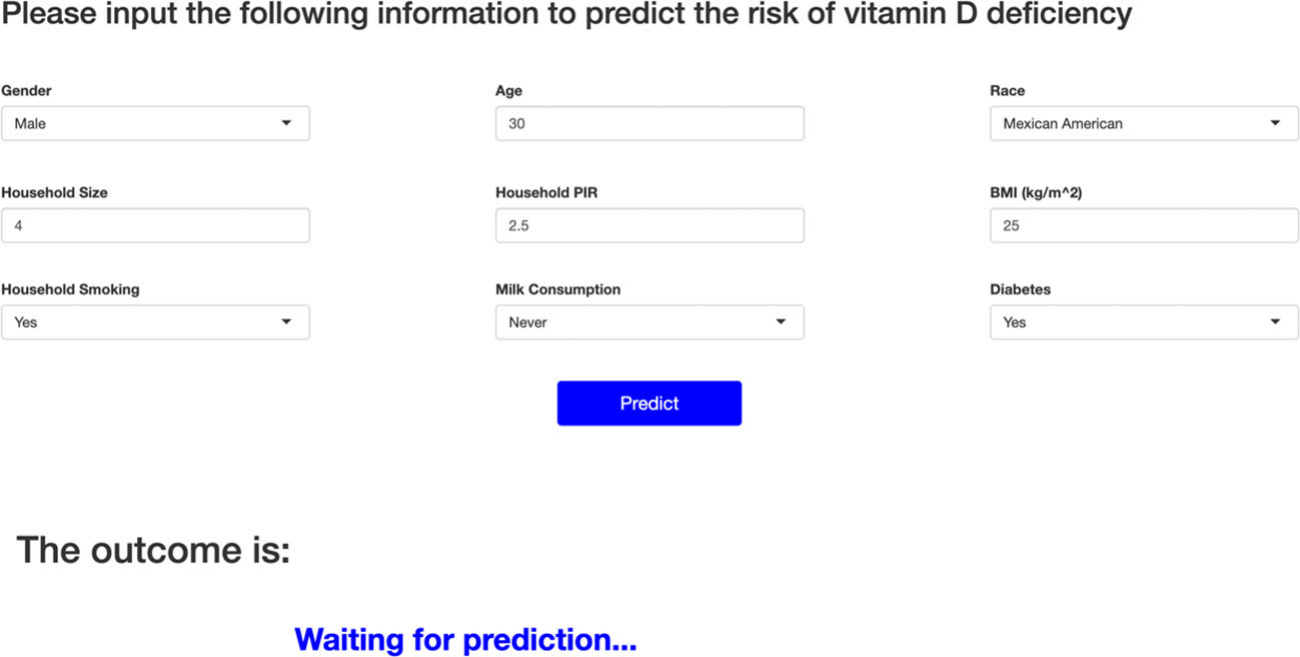
Online web calculator based on XGBoost modeling. Race is categorized as Mexican American, Non-Hispanic White, Non-Hispanic Black, Other Hispanic, or Other Race. Household Size: total number of people in the Household. Household Size over 7 or more defined as 7. Household PIR: household income to poverty income ratio. Household PIR more than 5 is defined as 5. BMI, body mass index; Household smoking, whether or not someone smokes in the household; Milk consumption, past 30-day milk product consumption. For Milk consumption, four frequencies were used to distinguish between never, rarely, sometimes, and often, with never meaning never drinking milk; rarely meaning less than once a week; sometimes meaning once a week or more but less than once a day; and often meaning once a day or more.

We further plotted a summary of SHAP values (**Figure 3**) to interpret the XGBoost model results. For each feature, a point corresponds to a patient. The position of the point on the X-axis (i.e., the actual SHAP value) indicates the effect of the feature on the model output for that particular patient.The higher the feature on the Y-axis, the more important the feature is to the model. The results show that for this model, the features included are, in order of importance, Race, Age, BMI, H.PIR, Milk, H.Size, Gender, H.Smoke, and Diabetes. We also constructed an online web calculator based on the XGBoost method in order to facilitate the use of the model (**Figure 4**, https://jialeguo.shinyapps.io/vitamin_D_deficiency/).

## 4 Discussion

This study uses data collected through interviews in a community-based population: gender, age, race, H.Size, H.PIR, BMI, H.Smoke, Milk, and diabetes. These nine variables were used as machine learning features to construct the model. Six machine learning methods (GBM, LR, NNet, RF, SVM, and XGBoost) were used to construct the model, and the model was evaluated for discrimination, fit, and clinical efficacy. **Figures 2A, B** show the main evaluation result of the discrimination: the ROC curve. The higher the convexity and the more skewed towards the upper left corner of the corresponding curve for each machine learning model, the better its differentiation. The results of the ROC curves in this study show that XGBoost-based has the best discrimination performance both in the training and validation sets. This is also confirmed in other complementary evaluation metrics: ACC, PPV, NPV, SEN, SPE, F1 score, and MCC. The results of the evaluation of clinical efficacy are presented in **Figures 2C, D**: DCA curves. The line corresponding to “Treat All” in the DCA curves shows the net benefit of intervening on all participants, and the line corresponding to “Treat None” shows the net benefit of not intervening on all participants. Therefore, it makes sense to construct a model that has a threshold probability that the net benefit is higher than both “Treat All” and “Treat None”. In this study, all the models have some clinical utility within a certain threshold. In particular, the model constructed by the XGBoost method has a higher net benefit than the “Treat All” or “Treat None” strategies within all thresholds. Ultimately, the model of the XGBoost method has the best and near perfect performance. This study further used SHAP values to interpret the model of XGBoost method, and among the variables included, race, age, and BMI were the top three important characteristics. In addition, an online web calculator was constructed based on the model of the XGBoost method for ease of use. Using this online web calculator, it is possible to screen community populations for vitamin D deficiency through a simple interview. The population in this study originated from the American community, where the prevalence of vitamin D deficiency was 32.11%. Vitamin D deficiency, a global public health problem, has different prevalence rates in different regions. Defined as vitamin D deficiency with 25(OH)D less than 50 nmol/L as recommended by an Endocrine Society Clinical Practice Guideline, the prevalence of vitamin D deficiency is 34.22% in Africa (25); 34.76% in South America (26); and 57.69% in Asia (27).

Both major forms of vitamin D forms (vitamin D2 and vitamin D3) are rarely found in food; vitamin D2 is found in plants and mushrooms; vitamin D3 is found in foods of animal origin, e.g., salmon, butter, and liver. Vitamin D in the body comes mainly from ultraviolet light exposure of the skin rather than through food intake. When human skin is exposed to ultraviolet light at wavelengths between 290 and 315 nm, it converts 7-dehydrocholesterol present in the epidermis to pre-vitamin D3 (28, 29). In turn, it is rapidly metabolized to vitamin D3 by thermal isomerization, which in turn is bound to vitamin D-binding proteins in the blood and transported to the liver. Converted to 1α,25(OH)2D3, the major biologically active metabolite form of vitamin D, sequentially by primary hydroxylation in the liver and kidney, respectively (28). This major source form of vitamin D in the body determines differences in vitamin D levels among different races and populations. The risk of vitamin D deficiency is related to race (30, 31), with darker-skinned races being less able to synthesize vitamin D from sunlight (32). In addition, latitude, season, atmospheric pollution, time spent outdoors, use of sunscreen, and habitual dress of some races, all factors that can affect the skin’s exposure to ultraviolet light, contribute to differences in vitamin D levels (32). The effect of age on vitamin D deficiency presents a different role in adults and minors. The results of a multicenter cross-sectional study of adults aged 30-75 years in Saudi Arabia suggest that older age is a protective factor against vitamin D deficiency (33). This has been confirmed in studies from other regions (34–36). Instead, for minors, a higher risk of vitamin D deficiency was predicted with increasing age (37, 38). Obesity increases the risk of vitamin D deficiency in different regions and ages (39–41). The results of a meta-analysis showed a positive association between BMI and vitamin D deficiency (42). Several Mendelian randomization studies have also demonstrated this relationship at the causal level (43, 44). Low vitamin D levels in the obese population may be caused by the deposition of vitamin D in the adipose zone of the body, which reduces its bioavailability (45).

Vitamin D plays a crucial role in the maintenance of calcium and phosphate homeostasis, normal bone growth and mineralization (46). The effect of vitamin D on mineral homeostasis is mediated by 1,25(OH)2 D activation of the vitamin D receptor (VDR) to stimulate intestinal calcium and phosphate absorption, renal tubular calcium reabsorption, and skeletal calcium mobilization (47). Vitamin D deficiency leads to decreased calcium and phosphorus absorption and lower circulating blood calcium, which is secondary to hyperparathyroidism. Parathyroid hormone (PTH) increases renal tubular calcium reabsorption and inhibits phosphorus reabsorption in order to maintain blood calcium levels (48), and ultimately, insufficient calcium phosphate products lead to systemic bone mineralization, causing rickets in children and osteomalacia in adults (49). Vitamin D is essential for bone health, and supplementation is essential for patients at risk for fractures and/or vitamin D deficiency (50). Besides roles closely related to calcium and phosphate homeostasis and bone metabolism, vitamin D has many roles to play, especially in the immune response. It can act directly on immune cells to promote an anti-inflammatory state and maintain the balance between pro- and anti-inflammatory (51). However, although vitamin D can affect the immune system in a number of ways, it tends to be interconnected with the microbiome and influence each other and the immune system (52). Vitamin D plays an important role in the immune response and maintenance of intestinal homeostasis by influencing the number and pathways of innate lymphoid cells (ILCs), which affect the immune response in the gut (53, 54). Recent studies have shown that the composition of the gut microbiota is altered by vitamin D levels (55, 56). The gut microbiota also influences calcium and vitamin D absorption, regulates intestinal permeability, hormone secretion and immune response (57). The intestinal epithelial VDR regulates autophagy and innate immune function through genes such as ATG16L1, which may influence the microbiota profile in the gut (58). Vitamin D deficiency also plays a key role in airway microbiome composition, as weekly oral supplementation has an impact on cystic fibrosis patients (59). Therefore, it is extremely important to use vitamin D and probiotics to regulate the immune system (60).

Prediction tools are widely used in the medical field to predict clinical disease diagnosis and prognosis. Several prediction tools have been used to predict vitamin D deficiency. However, there are no prediction tools for predicting the risk of vitamin D deficiency in the general community, including young people. In addition, the sample size included in this study far exceeds that of similar previous studies. The machine learning prediction tools developed by Sluyter et al. (61) are similar to ours: both are tools developed using data that could have been collected in the community through simple inspection and interviews. However, Sluyter et al.’s study was only applicable to adults older than 50 and performed worse than the XGBoost method in this study: the best AUC value for Sluyter et al.’s prediction tool was only 0.73; whereas the AUC value for the XGBoost method in this study was 0.995. Carretero et al. (62) and Kheir et al. (63) on the other hand developed prediction tools applied to hypertensive population and ICU admitted population respectively. Their AUC values were 0.74 and 0.64, respectively. This study is the first predictive tool that can be widely applied to predict vitamin D deficiency in community populations. The best performing XGBoost method in this study had perfect predictive performance. The large number of subjects is one of the strengths of this study, which resulted in the high accuracy of the results. The results of this study show that an online web calculator using the XGBoost method can be a good predictor of vitamin D deficiency in the general population. Using this predictive tool, screening for vitamin D deficiency in the community or primary care settings can be achieved at almost no cost, avoiding much of the public health expenditure on unnecessary vitamin D testing and providing an intuitive and powerful scientific tool for health education and further testing. Based on the results of the online web calculator in this study, primary care providers can give appropriate clinical advice to their patients and make timely interventions for those at high risk of vitamin D deficiency, especially for children, pregnant women, and the elderly.

However, we need to recognize that there are still some limitations to this study. First, in order for the predictor tool to be widely applicable to various scenarios, the vast majority of the predictors used in this study were based on participants’ self-reports, which may be subject to some bias. The NHANES database, on the other hand, has a strictly standardized process for data collection, and the large sample size of the studies included in this study can avoid these biases to a certain extent. Second, although internal validation was performed in this study by dividing the entire dataset into training and validation sets, we lacked external cohort studies to validate the performance of the prediction tool. All of the populations studied in this study were from the United States, and since vitamin D levels are related to factors such as race and latitude, the results of the study need to be viewed with caution when applied to populations in other regions. External validation of the study results using external datasets, especially from other continents, is necessary in the future.

## 5 Conclusion

The machine learning model constructed by the XGBoost method in this study possesses almost perfect performances. Based on this model, an online web calculator was further constructed, through which the risk of vitamin D deficiency in community populations can be predicted easily and quickly, and the public health expenditures caused by unnecessary vitamin D testing can be reduced.

## Data availability statement

Publicly available datasets were analyzed in this study. This data can be found here: Centers for Disease Control and Prevention (CDC), National Center for Health Statistics (NCHS), National Health and Nutrition Examination Survey (NHANES), https://wwwn.cdc.gov/nchs/nhanes/Default.aspx, NHANES 2001-2018.

## Ethics statement

The studies involving humans were approved by National Center for Health Statistics (NCHS) Research Ethics Review Board. The studies were conducted in accordance with the local legislation and institutional requirements. Written informed consent for participation in this study was provided by the participants’ legal guardians/next of kin.

## Author contributions

JG: Formal analysis, Methodology, Software, Visualization, Writing – original draft. QH: Software, Visualization, Writing – review & editing. YL: Supervision, Writing – review & editing.
